# Association between dose-volume parameters and acute bone marrow suppression in rectal cancer patients treated with concurrent chemoradiotherapy

**DOI:** 10.18632/oncotarget.21646

**Published:** 2017-10-06

**Authors:** Nan Li, Xue Liu, Fushan Zhai, Bing Liu, Xiaohui Cao, Shuyan Li, Minxian Zhang, Ming Liu

**Affiliations:** ^1^ Department of Radiotherapy and Oncology, Third Hospital of Hebei Medical University, Shijiazhuang, China

**Keywords:** concurrent chemoradiotherapy, acute bone marrow suppression, dose-volume parameters, rectal cancer, pelvic bone marrow

## Abstract

Concurrent chemoradiotherapy is one of the main treatments for rectal cancer. Bone marrow suppression is one of the critical factors that affect the progress of radiotherapy. We aimed to explore the association of incidence of acute bone marrow suppression with dose-volume parameters of pelvic bone marrow among rectal cancer patients with concurrent chemoradiotherapy. We retrospectively analyzed 50 rectal cancer patients for multivariate logistic regression analyses. Three subdomains of pelvic bone marrow (PBM), bilateral ilium (IBM), lower pelvis (LPBM), and lumbosacral spine (LSBM) were assigned. The radiation dose-volume parameters from the three subdomains and the whole pelvis were evaluated. Compared to Grade 0-1 leukopenia patients, ≥Grade 2 leukopenia patients exhibited significantly higher levels of IBM V_20_, V_25_, V_35_, mean dose (Dmean), LPBM V_20_, V_25_, V_30_, LSBM V_15_, PBM V_15_, V_20_, and PTV. The PBM V_20_ of ≥Grade 2 neutropenia patients was significantly higher than that of Grade 0-1 neutropenia patients. Multivariate analysis have demonstrated that IBM V_20_ and LSBM V_15_ were the independent factors affecting ≥ Grade 2 leukopenia. There is a correlation between low dose-volume parameters with acute bone marrow suppression. IBM V_20_, LSBM V_15_ and PBM V_20_ can be employed as the predictors of acute bone marrow suppression.

## INTRODUCTION

As a common malignant tumor, the incidence rate of rectal cancer had increased from 2000 to 2011 [1]. Currently, concurrent chemoradiotherapy is one of the main treatments for rectal cancer, which suppresses tumor local recurrence as well as improves overall survival [2, 3]. During concurrent chemoradiotherapy, Intensity Modulated Radiotherapy (IMRT) compares with Three-Dimensional Conformal Radiation Therapy (3-DCRT), IMRT exhibits dosimetric advantages in elevating conformality and dose gradient within target volume. IMRT significantly reduces organ-at-risk exposure [4, 5]. However, both radiotherapy and chemotherapy cause damages to bone marrow in different degree and loss of blood cells [6]. Hematological toxicity is a major cause of treatment interruptions which may lead to an increase in overall treatment time with a consequent detrimental effection. Bone marrow (BM), a primary hematopoietic tissue in humans, contains hematopoietic stem cells (HSCs), multipotent progenitors (MPPs), hematopoietic progenitor cell (HPCs), as well as multiple fully differentiated blood cells. Under normal physiological condition, HSCs are maintained in a quiescent state that is beneficial for lifelong hematopoiesis. Due to the short lifetime of granulocytes (6-8 hours), the first phenomenon of BM suppression is leukopenia(neutropenia). Irradiation to the BM cavity causes apoptosis and compromised proliferation of HSCs and progenitor cells. It also interrupts the resting state of HSCs, by inducing the cells from G0 phase into cell cycle, destroying the HSC niche [7]. Radiation not only induces BM suppression, but also directly kills granulocytes or causes chromosome alterations. The changes of microcirculation last for quite a long period. Because of their exuberant proliferation and low grade differentiation, BM and lymphoid tissues are extremely sensitive to radiation. Thus, the detrimental effects on BM are dependent on the dose, range, site and length of radiation. Hematopoietic bone marrow of healthy adults is mainly distributed in flat and irregular bones. More than 50% of hematopoietic bone marrow is found in hip, sacrum, proximal epiphysis of femur, and lumbosacral spine [8–9], all of which are within the irradiation range of radiation therapy for rectal cancer, inducing acute and chronic hematologic toxicity. Different drugs show remarkably different detrimental effects to bone marrow. Jin et al. [10] have found that leukopenia is the most severe side effect caused by capecitabine chemotherapy combined with radiotherapy in rectal cancer patients, since the incidences of Grade 2 and 3 leukopenia are 19.7% and 3.3%, respectively. It has also been documented that 69% rectal patients receiving concurrent chemoradiotherapy with capecitabine develop leukopenia, and 4% of the patients develop ≥ Grade 3 leukopenia [11]. It has been reported that only 4% of the colon cancer patients receiving capecitabine chemotherapy develop bone marrow suppression, indicating minimal side effects to bone marrow [12]. Therefore, we assumed that radiation therapy may be the leading cause of hematologic toxicity during concurrent chemoradiotherapy for rectal cancer.

In the present study, we analyzed the development of acute bone marrow suppression in rectal cancer patients receiving concurrent chemoradiotherapy, and explored the association between acute bone marrow suppression and general clinical factors as well as dose-volume parameters of pelvic bone marrow. Our study may provide potential predictors for bone marrow suppression.

## RESULTS

### Occurrence of acute bone marrow suppression

During the procedure of concurrent chemoradiotherapy, 37 patients (74%) developed acute bone marrow suppression, and the incidence of ≥Grade 2 bone marrow suppression was 28.0% (14/50) (Table 1)

**Table 1:** The occurrence of acute hematologic toxicity

	Grade				
Toxicity	0(%)	1(%)	2(%)	3(%)	4(%)
Leukopenia	21(42)	18(36)	10(20)	1(2)	0(0)
Neutropenia	38(76)	7(14)	4(8)	1(2)	0(0)
Anemia	38(76)	10(20)	0(0)	2(4)	0(0)
Thrombocytopenia	46(92)	2(4)	1(2)	1(2)	0(0)

Values are number (percentage).

### Analysis of clinical factors

The difference of gender, age, body mass index (BMI), clinical stage of the patients and surgery did not show significant impacts (P>0.05) on the occurrence of ≥ Grade 2 acute bone marrow suppression (Table 2)

**Table 2:** The relationship between clinical factors and incidence of bone marrow suppression (BMS) ≥Grade 2

Clinical factors	case(N)	The cases ≥Grade 2 acute bone marrow suppression		χ^2^ value	P value
		case(N)	incidence rate(%)		
Gender				0.043	0.551
male	31	9	29.03		
female	19	5	26.32		
Age				0.651	0.311
<60years	24	8	33.33		
≥60years	26	6	23.08		
BMI				0.021	0.446
<23.34	24	6	25		
≥23.34	26	8	30.77		
TNM stage				0.123	0.726
I-II	16	5	31.25		
III-IV	34	9	26.47		
Surgery				0.734	0.301
yes	31	10	32.26		
no	19	4	21.05		

BMI= body mass index; TNM= T:tumor, N:lymph node, M:metastasis.

### Univariate analysis of acute bone marrow suppression

**Figure 1:**
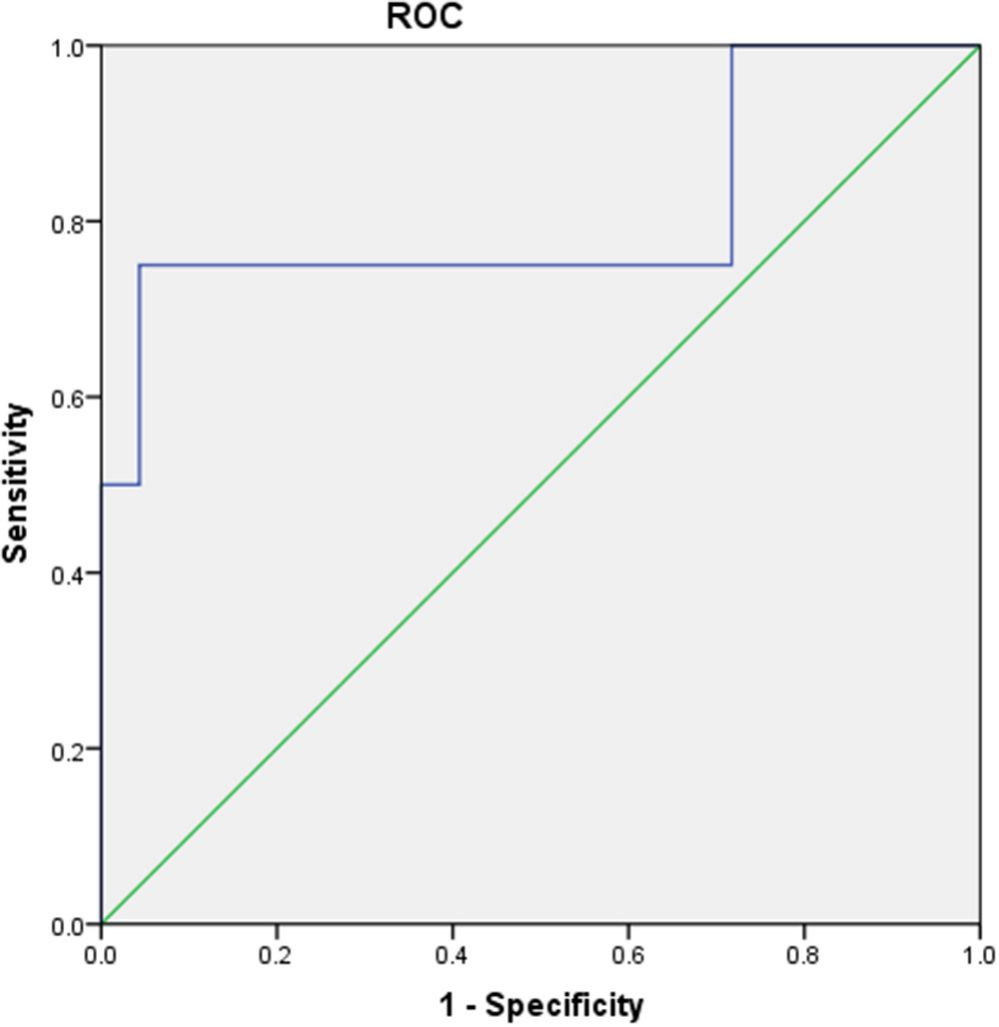
Receiver operating characteristic curve (ROC) of PBM V_20_ as predictor of ≥Grade2 neutropenia PBM V_20_ was significant associated with ≥Grade2 neutropenia (AUC=0.81, P=0.019). PBM= pelvic bone marrow; V_20_= the volume of bone marrow receiving 20 Gy or more.

Our data unveiled an association between leukopenia and dose-volume parameters of BM radiation. Compared to the patients with Grade 0-1 leukopenia, the patients with ≥ Grade 2 leukopenia showed a remarkable increase of several dose-volume parameters, including IBM V_20_, V_25_, V_35_, Dmean, LPBM V_20_, V_25_, V_30_, LSBM V_15_, PBM V_15_, V_20_, and PTV (P < 0.05) (Table 3). The PBM V_20_ of ≥ Grade 2 neutropenia patients was significantly higher than that of Grade 0-1 neutropenia patients (P < 0.05) (Table 4). ROC curve analysis demonstrated that the area under the curve (AUC) of PBM V_20_ was 0.81 and the predicted threshold was 83.59% (Table 5, and Figure 1 Receiver operating characteristic curve of (ROC) PBM V_20_ as predictor of ≥ Grade 2 neutropenia). However, there was no significant difference in the dosimetric data between ≥ Grade 2 and Grade 0-1 anemia and thrombocytopenia patients (P > 0.05).

**Table 3:** Grade 0~1 acute leukopenia VS. ≥ Grade 2 acute leukopenia dosimetry comparison

DVH parameters	acute leukopenia				t(z)	*P*
	Grade 0-1	range	≥Grade 2	range		
PBM						
V_15_%	74.8 (27.63)^*^	14.12-90.03	82(14.97)^*^	55.2-92.5	-2.073	0.038^°^
V_20_%	66.52(31.12)^*^	10.8-83.76	79(16.93)^*^	50.7-91.5	-2.471	0.013^°^
IBM						
V_20_%	54.41(29.38)^*^	0-83.29	67.5(12.96)^*^	43.1-80	-2.6	0.009^°^
V_25_%	39.9(20.98)^#^	0-76.46	54.59(13.61)^#^	26.1-78	-2.769	0.01^◊^
V_35_%	16(16.32)^*^	0-35.5	21.47(3.21)^*^	6.9-46	-2.12	0.043^°^
** Dmean(cGy)**	2098(1076)^*^	29-3193	2467(408)^*^	1805-3358	-2.166	0.03^°^
LPBM						
V_20_%	52.8(21.48)^#^	2.35-90.41	69.6(26.62)^#^	18-100	-2.173	0.035^◊^
V_25_%	39.39(19.58)^#^	0.99-72.58	55.42(26.13)^#^	14.4-100	-2.224	0.031^◊^
V_30_%	24.31(15.34)^#^	0.18-52.37	37.46(23.1)^#^	9.5-90	-2.233	0.03^◊^
LSBM						
V_15_%	87.17(36.44)^*^	0-100	97.87(12.3)^*^	86.4-100	-2.097	0.036^°^
**PTV (cm^3^)**	825.28(318.72)^#^	94.27-1690.65	1067.52(400.67)^#^	410.3-1796	-2.103	0.041^◊^

^◊^independent samples T test, ^○^Mann-Whitney U test.

^#^Data presented as mean percentage, with standard deviation in parentheses.

^*^Data presented as median percentage, with quartile range in parentheses.

PBM= pelvic bone marrow; IBM*=* iliac bone marrow; LPBM= lower pelvis bone marrow; LSBM= lumbosacral Spine bone marrow; V_15_, V_20_, V_25_, V_30_, V_35_= volume of bone marrow receiving 15, 20, 25, 30, 35Gy or more; DVH= dose-volume histogram; Dmean= the mean dose of iliac bone marrow; PTV= planning Target Volume.

**Table 4:** Grade 0~1 acute neutropenia VS. ≥ Grade 2 acute neutropenia dosimetry comparison

DVH parameters	acute neutropenia				Z	*P*
	Grade 0-1	range	≥Grade 2	range		
PBM V_20_(%)	67.35(25.04)	10.8-85	85.08(31.29)	50.73-91.5	-2.045	0.04

DVH= dose-volume histogram; PBM= pelvic bone marrow; V_20_= volume of bone marrow receiving 20Gy or more. Data presented as median percentage, with quartile range in parentheses.

**Table 5:** DVH parameters predictive ablity of ≥ Grade 2 acute leukopenia and ≥ Grade 2 acute neutropenia

DVH parameter	AUC	boundary value (%)	sensitivity (%)	specificity (%)	Asymptotic 95% Confidence Interval
IBM V_20_	0.759	61.09	0.91	0.67	0.616-0.902^▲^
LSBM V_15_	0.709	85.29	1	0.49	0.559-0.858^▲^
PBM V_20_	0.81	83.59	0.75	0.96	0.507-1^●^

^▲^acute leukopenia, ^●^acute neutropenia.

PBM*=* pelvic bone marrow; IBM= iliac bone marrow; LSBM= lumbosacral spine bone marrow; V_15_, V_20_= volume of bone marrow receiving 15, 20Gy or more; DVH= dose-volume histogram.

### Multivariate analysis of acute bone marrow suppression

**Figure 2:**
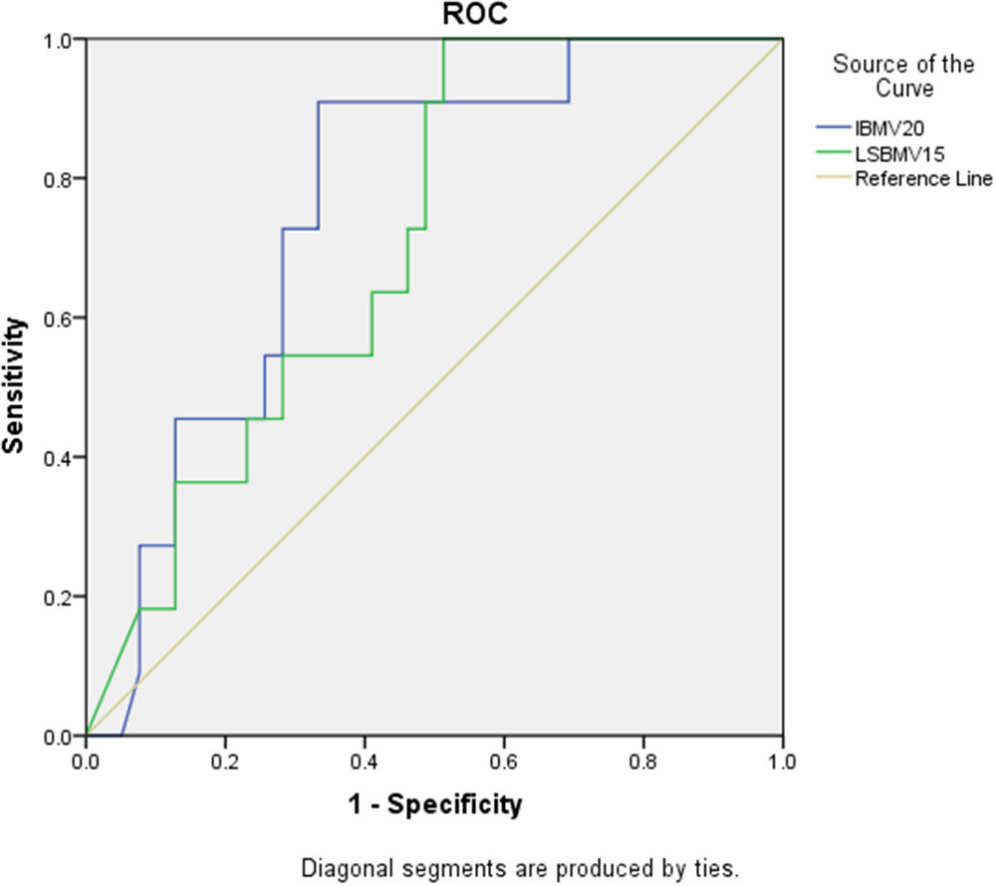
Receiver operating charicteristic curve (ROC) of IBM V_20_ and LSBM V_15_ as predictor of ≥Grade2 leukopenia The blue line is Iliac Bone Marrow V_20_ (AUC=0.81, P=0.03). The green line is Lumbosacral Spine Bone Marrow V_15_ (AUC=0.709, P=0.019). IBM V_20_ and LSBM V_15_ were significant associated with ≥Grade2 leukopenia. Abbreviations: IBM= Iliac Bone Marrow; LSBM = Lumbosacral Spine Bone Marrow; V_15_= The volume of bone marrow receiving 15Gy or more; V_20_= The volume of bone marrow receiving 20Gy or more.

The dose-volume parameters of statistical significance in the univariate analysis were selected for subsequent multivariate analysis, and the results demonstrated that IBM V_20_ and LSBM V_15_ were significant associated with acute leukopenia based on logistic regression model (P<0.05) (Table 6). IBM V_20_, LSBM V_15_, and PBM V_20_ were used for ROC curve analysis (Table 5). The AUC of IBM V_20_ and LSBM V_15_ were 0.759 and 0.709, and predicted thresholds were 61.09% and 85.29%, respectively (Figure 2 Receiver operating characteristic curve (ROC) generated by IBM V_20_ and LSBM V_15_).

**Table 6:** Logistic multifactor analysis of bone marrow DVH with ≥ Grade 2 acute leukopenia

DVH parameters	B	SE	Wald	*P*	Exp(B)
IBM V_20_	1.692	0.782	4.682	0.03	5.431
LSBM V_15_	0.903	0.386	5.458	0.019	2.467

Abbreviations: IBM= Iliac Bone Marrow; LSBM= Lumbosacral Spine Bone Marrow;V_15_, V_20_= volume of bone marrow receiving 15, 20Gy or more; DVH= Dose-volume histogram.

## DISCUSSION

Nowadays, concurrent chemoradiotherapy has been commonly employed for treatment of rectal cancer patients. Because of sensitization to radiotherapy caused by chemotherapy drugs, concurrent chemoradiotherapy has enhanced therapeutic outcomes. However, it should be noted that concurrent chemoradiotherapy is also accompanied with elevated damages to organs and overlaid hematopoietic toxicity, leading to increased incidence and extent of bone marrow suppression. Han et al. [13] and Duenas-Gonzalez et al. [14] have found that the incidence of ≥ Grade 3 bone marrow suppression is 40-60% in the cervical cancer and rectal cancer patients receiving concurrent chemoradiotherapy. It has been documented that the incidences of Grade 1-5 bone marrow suppression is 23%, 33%, 25%, 0%, and 0%, respectively, in the cervical patients during therapy [15]. Among the 50 patients enrolled in our study, the incidences of Grade 1-4 bone marrow suppression was 46.0%, 20.0%, 8.0%, and 0%, respectively, during the acute observation.

In a study involving 155 cervical cancer patients, Huang et al. [16] have reported that the development of bone marrow suppression is not significantly influenced by the general clinical factors, such as age, clinical stage, chemotherapy cycles, surgery, and methods of radiotherapy. Niu et al. [17] have retrospectively analyzed 99 patients with cervical cancer and concluded that bone marrow suppression is not associated with age, complications, clinical stage, tumor classification, chemotherapy regimen as well as course number of uterine artery infusion chemotherapy. The study of Mell et al. [6] has revealed that white blood cell nadir is correlated with female gender, low BMI and lymph node positivity, and neutrophil nadir is correlated with female gender and low BMI. Moreover, it has been reported that ≥ Grade 2 leukopenia is associated with cycle number of chemotherapy, and ≥ Grade 2 neutropenia is associated with cycle number of chemotherapy and T stage [18]. Single factor analysis in the present has demonstrated that acute bone marrow suppression is not significantly associated with gender, age, BMI and clinical stage of rectal cancer patients.

Bone marrow is considered a “parallel organ” in medical radiobiology. Thus, volume of radiation is directly related to the development of bone marrow suppression. Acute BM suppression is accompanied with irradiation induced apoptosis of HSCs and hematopoietic progenitor cells (HPCs) as well as disturbed proliferation of HPCs. Radiation not only induces BM suppression, but also directly kills granulocytes or causes chromosome alterations. The changes of microcirculation last for quite a long period. Mell et al. [8] has found that pelvis V_10_ is associated with ≥ Grade 2 leukopenia and neutropenia. The patients with pelvis V_10_ ≥ 90% are more susceptible to ≥ Grade 2 bone marrow suppression compared to those with pelvis V_10_ < 90%, while acute bone marrow suppression is not correlated with V_30_ and V_40_. A study involving 108 patients has demonstrated that pelvis D_max_ ≤ 57 Gy can reduce the occurrence of anemia, and pelvis V_10_ is associated with bone marrow suppression (threshold = 87%) [18]. Huang et al. [16] have retrospectively analyzed 155 patients and found that pelvis V_15_ is an independent risk factor of acute bone marrow suppression (threshold = 88%). Other studies have also unveiled the association between bone marrow suppression and low dose-volume parameters [19, 20].

Rose et al. have discovered that hematopoietic BM is mainly distributed in lumbosacral spine and pubis, while inactive BM is mainly distributed in ilium, ischium and proximal epiphysis of femur [20]. Using MRI and SPECT, Roeske et al. have found that hematopoiesis active domains are located in lumbosacral spine, middle ilium and iliac crest [21]. Thus, hematopoietic BM is not evenly distributed in the three sub-domains of pelvis: bilateral ilium, lower pelvis, and lumbosacral spine. Our data have revealed that ≥ Grade 2 leukopenia patients exhibit significantly higher levels of IBM V_20_, V_25_, V_35_, mean dose, LPBM V_20_, V_25_, V_30_, LSBM V_15_, pelvis V_15_, V_20_, and PTV compared to the Grade 0-1 leukopenia patients, and the PBM V_20_ of ≥ Grade 2 neutropenia patients is significantly higher than that of Grade 0-1 neutropenia patients, suggesting that ≥ Grade 2 bone marrow suppression is associated with low dose-volume of pelvis as well as each subdomain. Logistic regression modeling has demonstrated that IBM V_20_ and LSBM V_15_ are the independent factors correlated with ≥ Grade 2 leukopenia. ROC curve analyses of IBM V_20_, LSBM V_15_ and PBM V_20_ have determined their thresholds (61.09%, 85.29% and 83.59%, respectively), which may serve as optimal dosimetric thresholds.

PET-CT and MRI have been currently used for delineation of hematopoietic bone marrow and studies of bone marrow-sparing. MRI has been used to delineate hematopoietic bone marrow and to setup dose limitation, and the results have unveiled that the radiation dose to hematopoietic bone marrow V_5_ and V_10_ is significantly correlated to the incidence and severity of hematologic toxicity [22]. Liang et al. [23] have used PET-CT and MRI to delineate functional bone marrow and divided pelvic cancer patients into two groups: functional BM-IMRT group and total BM-IMRT group. There is a striking difference of functional bone marrow V_10_ and V_20_ between these two groups, indicating a better bone marrow protection can be achieved by singularly limiting dose of functional bone marrow instead of total bone marrow.

In conclusion, acute bone marrow suppression in the rectal patients receiving concurrent chemoradiotherapy is significantly associated with decreased dose-volume parameters. Three independent risk factors, IBM V_20_, LSBM V_15_, and PBM V_20_, may be employed to predict the occurrence of acute bone marrow suppression when designing therapeutic plans. Next, we will carry out perspective studies on the development of bone marrow suppression after setting up protective limitations of PBM radiation volume.

## MATERIALS AND METHODS

### Patients

We retrospectively analyzed 50 patients with rectal cancer treated at the Department of Oncology of the Third Hospital of Hebei Medical University from January 2010 to March 2016, and collected clinical data regarding blood cell numbers, diagnosis and treatment. The patients diagnosed with rectal cancer (KPS ≥ 70) and receiving concurrent chemoradiotherapy were included in this study, while patients with discounted radiotherapy, pre-existing bone marrow suppression before treatment, or bone metastases were excluded. There were 31 male and 19 female patients with a mean age of 59 (range, 21-78 years). Patients were classified according to the Union for International Cancer Control (UICC) TNM staging system (7^th^ edition), and the pathological type of all the tumors was adenocarcinoma.

### Radiotherapy

All patients were in the supine position and immobilized with Med-Tech thermoplastic sheets. Simulation and positioning were performed with Somatom-sensation Plus-16 spiral CT scanner (Siemens, Germany) and LAP laser system, and the treatment plans were designed using CMS-Xio4.4 planning system (USA). Five- or seven-field plan was chosen in the intensity modulated radiation therapy (IMRT). The photon energy of X-ray irradiation was 6 MV. Dose to 95% (D95) of planning target volume (PTV) ranged between 45 to 62 Gy (median dose was 48Gy), and the radiation was performed at 1.8-2.0 Gy daily, 5 times/week, 5-6 weeks. All the patients were treated with clinical linear accelerator (Varian-IX, USA).

### Delineation of target volumes

The gross tumor volume (GTV) included the primary tumor lesions and metastatic lymph nodes. The clinical target volume (CTV) consisted of the GTV and regional draining lymph nodes, including mesentery of rectum, presacral space, iliac blood vessels, and ischiorectal fossa, with the upper bound between L5 and S1 and the lower bound at 2 cm below the inferior margin of rectal lesion. Ischiorectal fossa was only included when cancer lesion were found in lower rectum. The planning target volume (PTV) was obtained by expanding CTV with a 0.5-1.0cm margin to account for set up uncertainty and organ motion.

### Delineation of organs at risk (OAR)

**Figure 3:**
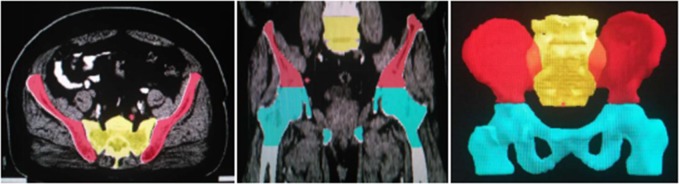
2D and 3D pictures of contour of pelvic bone The red zones are bilateral ilium, the light blue zones are lower pelvis, and the yellow zone is lumbosacral spine.

OAR was delineated according to the guidelines for the delineation provided by the Radiation Therapy Oncology Group (RTOG), including the bladder, the intestine within irradiation range, femoral head, and testicles. We delineated pelvic bone marrow (PBM) to replace hematopoietic bone marrow as first described by Mell et al.[8]. The entire PBM was divided into three subdomains: (1) bilateral ilium bone marrow (IBM)—the iliac crest extending to the upper border of femoral head; (2) lower pelvis bone marrow (LPBM)— the region extending from the superior border of femoral head to the inferior border of ischial tuberosities, including pubes, ischia, acetabula, and proximal femora; (3) lumbosacral spine bone marrow (LSBM)— the region extending from the superior border of L5 to coccyx. (Figure 3 2D and 3D pictures of contour of pelvic bone).

### Concurrent chemoradiotherapy protocol

All the patients were given capecitabine orally twice a day (1650 mg/m^2^/d) for 5 days every week simultaneously with radiotherapy.

### Collection of pelvic dose-volume parameters

**Figure 4:**
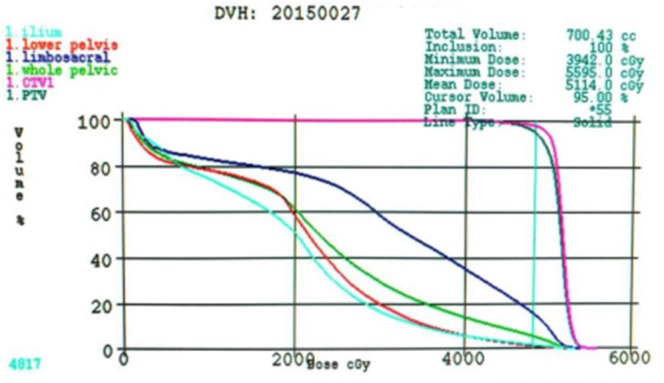
DVH of IMRT plan The light blue line is ilium, the red line is lower pelvis, the dark blue line is limboscacral, the green line is whole pelvis, the pink line is CTV1 and the dark green line is PTV. Abbreviations: DVH= Dose-volume histogram; IMRT= Intendity-modulated radiotherapy.

The volumes of IBM, LPBM, LSBM, and PBM receiving 10, 15, 20, 25, 30, 35, and 40Gy (V_10_, V_15_, V_20_, V_25_, V_30_, V_35_, and V_40_) were quantified. The average dose(Dmean) as well as PTV dose and volume were also analyzed. (Figure 4 DVH of IMRT plan).

### Grading of acute bone marrow suppression

We set the initiation and finish of radiotherapy as the starting point and end point of this study. Blood routine examinations were carried out before the initiation of radiotherapy to exclude the patient with pre-existing bone marrow suppression. During the concurrent chemoradiotherapy, we collected complete blood count weekly. Acute bone marrow suppression was graded according to the RTOG acute radiation morbidity scoring criteria [24].

### Statistical analyses

Statistical analyses were performed using SPSS 21.0 software. Quantified data were analyzed by chi-square test or Fisher's exact test. The mean values of two normally distributed samples were compared using t test, and two independent samples without normal distribution were modeled using multivariate logistic regression for screening independent factors. Receiver operating characteristic curve (ROC) analysis was performed to determine the bone marrow dosimetric thresholds of acute bone marrow suppression.

## Abbreviations

UICC: Union for International Cancer Control
IMRT: intensity-modulated radiotherapy
BMS-IMRT: bone marrow sparing intensity-modulated radiation therapy
CT: computer tomography
GTV: gross tumor volume
CTV: clinical target volume
PTV: planning target volume
OAR: organ at risk
PBM: pelvic bone marrow
IBM: iliac bone marrow
LPBM: lower pelvis bone marrow
LSBM: lumbosacral spine bone marrow
V_10_: the volume of bone marrow receiving 10 Gy or more
Dmean: the mean dose of bone marrow
DVH: dose-volume histogram
WBC: white blood cell
ANC: absolute neutrophil count
HGB: hemoglobin concentration
PLT: platelet
BMI: body mass index
ROC: receiver operating characteristic curve
AUC: area under curve
TNM: T: tumor, N: lymph node, M: metastasis
